# Microbiological Analysis of Wild Lowbush Blueberries Harvested in Nova Scotia, Canada for the Fresh Produce Market

**DOI:** 10.3390/microorganisms12112251

**Published:** 2024-11-07

**Authors:** Timothy Ells, Nancy Tregunno, Lihua Fan, Michele Elliot, Craig Doucette, Hugh Lyu, Alexa Jollimore

**Affiliations:** 1 Agriculture and Agri-Food Canada Kentville Research and Development Centre, Kentville, NS B4N 1J5, Canada; 2 Perennia Food and Agriculture Inc., Bible Hill, NS B6L 2H5, Canada

**Keywords:** wild lowbush blueberries, fresh-packed produce, minimal processing, fecal indicators, *Listeria monocytogenes*

## Abstract

Canada is a leading producer of wild lowbush blueberries, most of which are mechanically harvested, washed, individually quick frozen (IQF), and bulk packaged. Still, some berries are harvested by more gentle methods and sold as fresh-packed products. These berries do not undergo a wash step, nor are subjected to antimicrobial treatments. The purpose of this study was to conduct a microbiological survey of berries harvested in the province of Nova Scotia to assess their potential for harborage of bacterial foodborne pathogens. A combination of standardized plate count methods and 3M-Petrifilm protocols were used to enumerate total aerobic mesophilic bacteria (APC), yeasts and molds (YMC), coliforms, and generic *E. coli*, the latter being an indicator of fecal contamination. Overall, APC and YMC levels were 1.2 and 0.5 log greater, respectively, for berries collected early in the harvest season versus those acquired late season and varied significantly (*p* < 0.05) between farm (location) and harvest practices used. Berries harvested by our team using sanitized hand rakes (SH) had consistently lower APC and YMC levels than those harvested by farm crews. Yet, when gentle harvesting (GH) methods (hand-raking, walk-behind or modified mechanical harvesters) were employed on farms, lower numbers were generally observed compared to berries harvested by traditional tractor-mounted mechanized harvesters (MH). The presence of coliforms (and their levels) was also impacted by the harvest method, with detection rates of ~29%, 73%, and 92% in SH, GH, and MH samples, respectively. Mean counts were < 2.5 log_10_ CFU/g for both SH and GH berries, but significantly higher (*p* < 0.05) on MH berries (3.6 log_10_ CFU/g). Although ~56% of all berry samples collected (n = 350) contained coliforms, only 12 were positive for *E. coli*, 9 of which were MH samples. Only the latter had numbers > 2 log_10_ CFU/g, but none tested positive for Shiga toxin-producing serotype O157 (STEC O157) or *Salmonella* spp. when using internationally recognized selective enrichment and plating methods. ATP luminescence was used to assess the general hygiene of processing lines, whereby “hot spots” for microbial activity were identified, even after cleaning., Standard selective enrichment and plating methods were used for the detection of *Listeria monocytogenes* on 61 swab samples taken from berry totes or conveyor belts at different times during processing; 4 swabs tested positive for *L. monocytogenes*. However, the pathogen could not be detected by direct plating on selective agar without prior enrichment; this indicated its numbers were low. The results from this work demonstrated that alternative gentle harvest methods can reduce microbial numbers on wild blueberries. Although the frequency of fecal contamination in berry samples appeared to be low and targeted human pathogens were not detected; this represents a single study conducted over one harvest season. Therefore, it would be prudent for processors to seek effective antimicrobial technologies prior to packaging, while consumers should use caution and thoroughly wash produce before consumption. Where sporadic detection of *L. monocytogenes* was observed on environmental samples from the processing line, processors must ensure that effective sanitation programs are implemented to avoid potential food safety risks.

## 1. Introduction

The consumption of berries (e.g., blackberries, blueberries, raspberries, and strawberries) offers well-recognized health benefits due to their elevated levels of bioactive compounds [1]. Despite their health-promoting attributes, berries (fresh or frozen) can pose a risk for the transmission of foodborne disease [2]. Soft fruits such as berries are most often consumed raw without undergoing treatments aimed to destroy enteric pathogens and their toxins. Moreover, for berries destined for the fresh fruit market, minimal handling is desired so as not to compromise their physical integrity. Therefore, these berries are normally packaged without a washing step since wetting can promote mold growth, thereby shortening their shelf life [3]. Consequently, an increasing number of reported outbreaks of foodborne illnesses have been linked to the consumption of fresh and frozen berries.

In 2020, a report was published by the Institute of Food and Agricultural Services at the University of Florida (i.e., UF/IFAS Extension) that identified numerous events where various berries, or combinations of mixed berries, had been linked to foodborne diseases [4]. The same report also listed an additional five outbreaks where berries were considered the probable cause. Most of these outbreaks involved raspberries (15) or strawberries (12) individually, or mixtures containing these berries (10), while other outbreaks involved contaminated blackberries (5) and blueberries (2). Although a wide variety of foodborne pathogens (i.e., bacteria, fungi, parasites, and viruses) were responsible for these outbreaks, half were caused by the ingestion of the hepatitis A virus (HAV) or norovirus (NoV), while another 15 resulted from the microscopic parasite, *Cyclospora cayetanensis* [4]. Tavoschi et al. (2015) also published a report focused solely on European events between 1983 and 2013 (outbreaks or contamination recalls) involving berries [5]. Of the 32 independent incidents reported, 26 occurred after 2004 and in all but one case, NoV or HAV were the infectious agents.

The microbiological quality of berry crops can be impacted by a number of intrinsic and extrinsic factors [6]. The type of berry, the weather conditions during their growing and harvest seasons, and the geographical location of fields or plots influence the number and types of resident microflora on the berries. In addition, harvest practices and post-harvest processing/handling will also play a role in the microbiological status of the berries. As such, foodborne pathogens may be present among these microbial constituents and contaminate berries during pre-harvest production, at the time of harvest, or during processing. The risk is the greatest for commodities that grow in close proximity to the ground as berries may come into contact with soil debris. The most probable source for contamination of berries with human pathogens is through contact with fecal matter (animal and human). Wildlife such as bears, coyotes, deer, rabbits, raccoons, and flocks of various bird species frequently enter production plots or fields where crops grow [7]. In addition, contaminated irrigation water [8], run-off water from pastures supporting livestock [9], and poorly composted manures [10] can pose a potential risk for pathogen transfer. Worker hygiene has also been implicated as a major source of contamination for events associated with outbreaks and/or recalls involving berry crops [6].

Canada is a global leader in the production and export of wild (i.e., lowbush) blueberries (*Vaccinium angustifolium* Ait). With 65 million hectares of commercially managed lands and annual yields of over 135 million kg, wild blueberries are Canada’s most valuable fruit export with an estimated value of a quarter of a billion dollars [11]. Although wild blueberries grow naturally in all regions across the country, over 90% of commercially harvested fruit comes from the Saguenay-Lac-Saint-Jean region of the province of Quebec and the maritime provinces of New Brunswick, Nova Scotia, and Prince Edward Island. Canada’s most eastern province Newfoundland and Labrador also contributes to the commercial harvest, but its annual volumes are substantially less (~140 thousand kg) than those of the aforementioned provinces [12].

For Nova Scotia, wild blueberries represent the province’s most important agricultural export with ~30 million kg harvested and processed each year. Presently, traditional tractor-mounted harvesters are the most efficient equipment used to harvest the majority of the crop, which are then processed and generally sold as frozen products. Although individual steps can vary depending on the processor, the berries usually go through the following stages: (1) winnowing after harvest to remove large debris (leaves, twigs, soil clumps, etc.), (2) fresh water wash to remove fine particulates, (3) some processors use a sugar flotation step to separate green berries (sink) from ripe (float), (4) chlorinated-water spray for disinfection, and finally, (5) the berries are individually quick-frozen (IQF) and packaged (format depending on the end user), with the bulk of berries destined for export to foreign secondary processors [13]. The financial returns for these berries are subject to commodity wholesale pricing. In contrast, each year 2–3% of Nova Scotia’s wild blueberry crop is channeled to the more lucrative fresh fruit market where growers may see a >200% increase in value relative to their IQF counterparts [11].

Since wild blueberries grow near the ground, they are likely to contain relatively high levels of soil or debris-associated microflora, especially when machine-harvested. Hazen et al. (2010) reported that IQF processing (washing, chlorinated spray, and freezing) reduced the overall load of aerobic mesophilic bacteria by more than 2 log on lowbush blueberries harvested in Maine [13]. Prior to processing, approximately 66% of the samples taken in this study contained coliforms, ~36% of which tested positive for *E. coli*. However, coliform bacteria were present in only a single sample among the 24 taken after processing and none contained *E. coli*, thereby demonstrating the efficacy of these processing steps with respect to microbiological quality and safety.

On the other hand, blueberries chosen for the fresh-packed produce market receive special attention during handling, where they are harvested by more gentle means such as hand-raking or by modified machine harvesters deployed on selected fields with flat terrain which lessens contact with soil and debris. These blueberries usually do not undergo a washing step as submersion or rinsing in water can remove the protective natural bloom on the berries and residual water will promote the growth of molds [3]. Instead, cleaning often involves passing the berries through a “blower” to remove debris and fine particulates, after which the berries are sorted and packaged without antimicrobial interventions. Therefore, questions remain regarding the microbiological safety of wild blueberries sold as fresh-packed ready-to-eat (RTE) products as risk assessment data are unavailable. This represents a serious knowledge gap for the local industry.

Wild blueberry producers in Nova Scotia would like to maximize their financial returns by sending a greater portion of berries to the fresh fruit market. However, given that the processing of fresh-packed blueberries omits the typical IQF processing steps known to reduce microbial loads, growers require more information regarding the safety of these products. While several surveillance studies have been conducted for a variety of foodborne pathogens on other fruit commodities, including frozen berries such as strawberries and raspberries [14], a limited number have focused on either fresh-packed or frozen wild blueberries [15,16]. Specifically pertaining to Nova Scotia wild blueberries, microbiological studies have not focused on food safety issues but instead have concentrated on plant diseases and the soil or rhizosphere microbiomes [17,18].

The primary objective of the present study was to conduct a microbiological survey to assess the potential carriage of bacterial foodborne pathogens on Nova Scotia wild blueberries. Given the fact that growers employ more gentle harvesting methods for berries selected for the fresh-pack market, and these berries are not washed or subjected to anti-microbial treatments, this study examined the impact these alternative harvest methods have on the microbiological status of berries entering the processing lines, with particular focus on the potential carriage of fecal contaminants and bacterial foodborne pathogens. Providing such information will allow growers to have science-based information to make decisions that could help avert potential food recalls or outbreaks of foodborne diseases which could ultimately damage the reputation of the industry and impact international trade. The results of this study are presented herein.

## 2. Materials and Methods

### 2.1. Blueberry Sample Collection

A total of 350 individual pre-processed blueberry samples were collected in duplicate during the 2021 harvest season running from early August to early September (five to six weeks). Samples were collected from Monday to Wednesday during each week to allow time for the berries to be processed in the laboratory. Sample collection was limited to dry days to avoid wetting of the berries. An individual sample constituted a one-pint vented polyethylene-terephthalate (PET) clamshell pack. To discern the impact of harvest methods on the microbiological status of the berries, three sample types were partitioned according to the method used (i.e., SH, sanitized hand-rake/harvested by the research team; GH, gently harvested by farm crews by either hand-raking, walk-behind harvester or modified mechanical harvester; MH; mechanically harvested by traditional mechanical tractor-mounted harvester). Sample types were further delineated by the farm (five operations), fields, and time of the season (early or late). Table 1 gives a breakdown of the number of samples collected prior to processing. Field samples collected by the research team were taken from five random locations in each selected field. For this “sanitary” harvesting (SH) procedure, hand rakes were surface-sterilized by misting with a chlorinated (100 ppm) disinfectant spray and then wiped dry with a clean paper towel before collecting each sample. Raked berries were sieved through a metal grate held over a clean tote, allowing berries to pass through while separating larger debris such as leaves and twigs with mechanically assisted air movement. Berries gently harvested by farm personnel (GH) were obtained by collecting samples entering the blower on the sorting line at each processing facility. Mechanically harvested (MH) berries were collected directly from plastic totes in the fields where machines were operating or upon arrival at packing sheds where they were collected from totes at different times during the day to ensure sample diversity in terms of fields or locations within fields. Once clamshell containers were filled with berries, they were immediately placed into coolers containing ice packs. If the samples could not be returned to the laboratory in the evening of each sampling day, coolers were moved to a walk-in cold room (4–5 °C) before being transferred to the laboratory the next day. Processing of all samples for microbiological analyses occurred within 48 h of their collection (except for heat-resistant mold enumerations). The duplicate sample packs were immediately placed in a freezer upon arrival at the laboratory.

### 2.2. Microbiological Analysis

#### 2.2.1. General Quality and Spoilage Potential

Culture-based microbiological methods were employed to enumerate total aerobic bacteria (APC, aerobic plate count), yeasts and molds (YMC) as well as heat-resistant molds (HRM). For each individual sample (i.e., clamshell pack), ~25 g of berries was placed in a 400 g filtered stomacher bag and diluted 10-fold with pre-chilled (4 °C) buffered peptone water (BPW) using a DiluFlow gravimetric diluter (Interscience Inc., Woburn, MA, USA). The samples were homogenized for two minutes on the normal speed setting in a Seward stomacher blender (West Sussex, UK) and then immediately placed in a cooler to limit any growth of resident microorganisms prior to serial dilutions and subsequent plating. To achieve general microbial counts for SH, GH, and MH berries representative of each of the fields or berry lots sampled, the five individual samples collected from each location were combined into a single sample following homogenization. Specifically, this was performed by combining 5 mL of the above homogenates from each of the five associated samples (from a single field or lot) in a sterile disposable 50 mL conical centrifuge tube (e.g., the five early season samples taken from field #1 of Farm #1 were combined). The composite samples (70 in total) were thoroughly vortexed, serially diluted accordingly in 0.1% peptone water, and then spiral plated (Don Whitley Scientific Ltd., West Yorkshire, UK) onto Plate Count Agar (PCA) for APCs, whilst for YMCs, potato dextrose agar (PDA) supplemented with 100 µg/mL chloramphenicol was used to inhibit the growth of competing bacteria. The plates were incubated at 30 °C for 48 h and 25 °C for 5 days for APC and YMC, respectively.

#### 2.2.2. Fecal Contamination

Coliforms and *Escherichia coli:* To assess the potential risk for carrying human pathogens, coliform bacteria, and *E. coli* counts, respectively, were used as indicators for general hygiene and fecal contamination. All individual sample homogenates from above (n = 350) were screened using 3M™ Petrifilm™ *E. coli*/Coliform Count Plates (3M Canada, London, ON, Canada). One milliliter of initial berry homogenate or dilutions were applied to the film and incubated according to the manufacturer’s directions. Samples displaying no coliform growth were then re-plated (5 mL) onto 3M™ Petrifilm™ High-Sensitivity Coliform Count Plates (3M Canada, London, ON, Canada) using the recommended protocol provided by the manufacturer.

#### 2.2.3. Detection of Pathogens: *E. coli* O157 and *Salmonella* spp.

All samples that tested positive for the presence of *E. coli* on the PetriFilm™ were further screened for Shiga toxin-producing serotype O157 (STEC O157) and *Salmonella* spp. For STEC O157, 25 g of berries from the *E. coli*-positive samples (Section 2.2.2) were homogenized as described above except modified tryptic soy broth (Oxoid Canada, Nepean, ON, Canada) supplemented with 20 µg/mL novobiocin (mTSB-n) was used in place of BPW to provide a selective advantage for the O157 serotype [19]. Stomacher bags containing the homogenates were incubated at 41.5 °C for 24 h. Following incubation, 1 mL of this enrichment culture was subjected to immunomagnetic separation using Dynabeads^®^ Max anti-*E. coli* O157 (Life Technologies Corporation, Frederick, MD, USA) following the manufacturer’s instructions to concentrate the target pathogen. The separated Dynabeads^®^ were resuspended in 100 µL of phosphate-buffered saline containing Tween^®^ and vortexed vigorously to dislodge captured bacteria. Subsequently, 10 µL aliquots were streaked onto cefixime tellurite sorbitol-MacConkey agar containing 5-bromo-4-chloro-3-indolyl-b-d-glucuronide (CT–SMAC-BCIG, Oxoid) and plates were incubated at 35 °C for 24 h. To screen for *Salmonella* spp. 25 g of berries were pre-enriched in 3M™ *Salmonella* enrichment broth (3M Canada, London, ON, Canada) at 41.5 °C for 24 h. A 0.1 mL aliquot of pre-enrichment culture was then transferred to 10 mL of Rappaport-Vassiliadis R10 broth (RLM; Bio-Rad Laboratories, Hercules, CA, USA) and incubated for 24 h at 41.5 °C to select for salmonellae. Following incubation, 10 µL of culture was streaked onto hydrated 3M™ Petrifilm *Salmonella* Express plates (SALX, 3M Canada, London, ON, Canada) according to the manufacturer’s directions and incubated at 41.5 °C for 24 h.

#### 2.2.4. Enumeration of Heat-Resistant Molds (HRM)

HRM numbers were determined using the pour plate method [20,21] with modifications. In brief, each 200 g blueberry sample had a composite of 40 g of fruit from each of the five clamshell samples collected from the same field or sample lot (n = 70). Each sample of berries was homogenized with 120 mL of sterile distilled water for two minutes in a laboratory blender. The sample was then divided into two 500 mL media bottles and then placed in a water bath. One bottle was fitted with a thermocouple to monitor the temperature of the heat-treated samples. When the temperature reached 80 °C the samples were held for 30 min and then 100 mL of molten, double strength, Potato Glucose Rose Bengal agar (PGRB) (Honeywell Fluka, Charlotte, NC, USA) was added to each bottle and thoroughly mixed. The treated samples from the two bottles were cooled to ~50 °C before being distributed into eight Petri dishes (12 cm × 12 cm × 1.5 cm) (L × W × H). Samples were incubated at room temperature for 10 days before enumeration of HRM which were expressed as log_10_ CFU/kg.

### 2.3. Environmental Sampling

#### 2.3.1. General Sanitary Assessment

As an indicator of the general cleanliness of the conveyor belt surfaces after routine cleaning, adenosine triphosphate (ATP) levels were measured using the 3M™ CleanTrace™ system (3M Canada, London, ON, Canada). Surfaces (~100 cm^2^) were sampled using 3M™ UXL100 swabs (3M Canada, London, ON, Canada). ATP activity on the activated swabs was measured using a 3M™ Clean-Trace™ luminometer (3M Canada, London, ON, Canada) and the output was recorded as relative light units (RLU). Swab samples from the conveyor belts were taken at four different locations on the processing line (i.e., receiving, tilt, sizer, and inspection belts) and at different times during the harvest season (early, middle, or late).

#### 2.3.2. Detection of *Listeria monocytogenes* on Berry Contact Points

Since *L. monocytogenes* is known to be problematic as a persistent colonizer of surfaces across the RTE food industry, its presence on food contact surfaces within the participating blueberry processing facilities was monitored. Here, 3M™ sponge sticks (3M Canada, London, ON, Canada) were used to sample the conveyor belts and field totes (before routine cleaning). A total of 31 sponge swab samples were taken from conveyor belts along the processing line and another 30 were taken from emptied berry totes. The pre-wetted sponges were rubbed along test surfaces (~100 cm^2^) as directed by the manufacturer and then returned to their zip-locked bags. The samples were placed in portable coolers containing ice packs and then transported to the laboratory. To process samples, each sponge was released from its stick-holder directly into a stomacher bag containing 40 mL of phosphate-buffered saline (pH 7.2). Each sample was homogenized in a compact IUL Masticator blender (IUL S.A., Barcelona, Spain) for 1 min at room temperature. The sponge was raised above the surface level of the buffer within the stomacher bag and then squeezed to ensure maximum recovery of liquid. After aseptically removing the sponge from the bag, 20 mL of the homogenate was transferred to a 50 mL conical centrifuge tube. To this, sterile glycerol was added and mixed to achieve a final concentration of 20% *w*/*v* and the tube was then stored at −80 °C. Detection of *Listeria* was carried out using the International Organization for Standardization (ISO) method, EN 11290-1:2017, with a slight modification [22]. Specifically, 90 mL of half Fraser broth was added to the stomacher bag containing the remaining 10 mL of sample homogenate, gently mixed, and then incubated for 24–48 h at 30 °C. After incubation, the ensuing steps of ISO 11290-1:2017 were followed [22]. Samples that turned black in the Fraser broth within 48 h of incubation were streaked onto both modified Oxford agar and Rapid L’mono agar (RLM; Bio-Rad Laboratories, Hercules, CA, USA). The plates were incubated at 35 °C for 48 h, and then assessed for the presence of colonies displaying typical *L. monocytogenes* morphology.

#### 2.3.3. Enumeration of *Listeria monocytogenes*

For enrichment samples that tested positive for *L. monocytogenes*, the preserved homogenates previously stored at −80 °C were thawed. It was anticipated that the samples would contain low levels of *Listeria* since the development of characteristic black precipitate during enrichment was slow. To accommodate the predicted low levels of *Listeria* in the samples, 200 µL of undiluted homogenate was directly spiral plated onto each of five plates of modified Oxford agar. The plates were then incubated at 35 °C for 48 h and colonies displaying typical morphology for *Listeria* spp. colonies were enumerated. Presumptive *Listeria* spp. colonies were verified as *L. monocytogenes* by streaking onto RLM.

### 2.4. Statistical Analysis

Two-way analysis of variance (ANOVA) was used to compare APC, YMC, HTM, and coliform counts delineated by time within harvest season (early versus late) and harvest method (GH, MH, SH) as factors. One-way ANOVA was used to compare microbial counts on berries harvested by each method on each participating farm and for analysis of all berries harvested by a particular method regardless of farm location or harvest season. All pairwise multiple comparison procedures were conducted using the Holm–Sidak method and results were considered significant at *p* < 0.05. The statistical package within SigmaPlot v 15 software (Systat Software Inc., San Jose, CA, USA) was used to perform the ANOVA analyses.

## 3. Results

### 3.1. Total Aerobic Bacteria, Yeast and Molds, and Heat-Resistant Molds

#### 3.1.1. Impact of Harvest Method and Harvest Season

**Figure 1:**
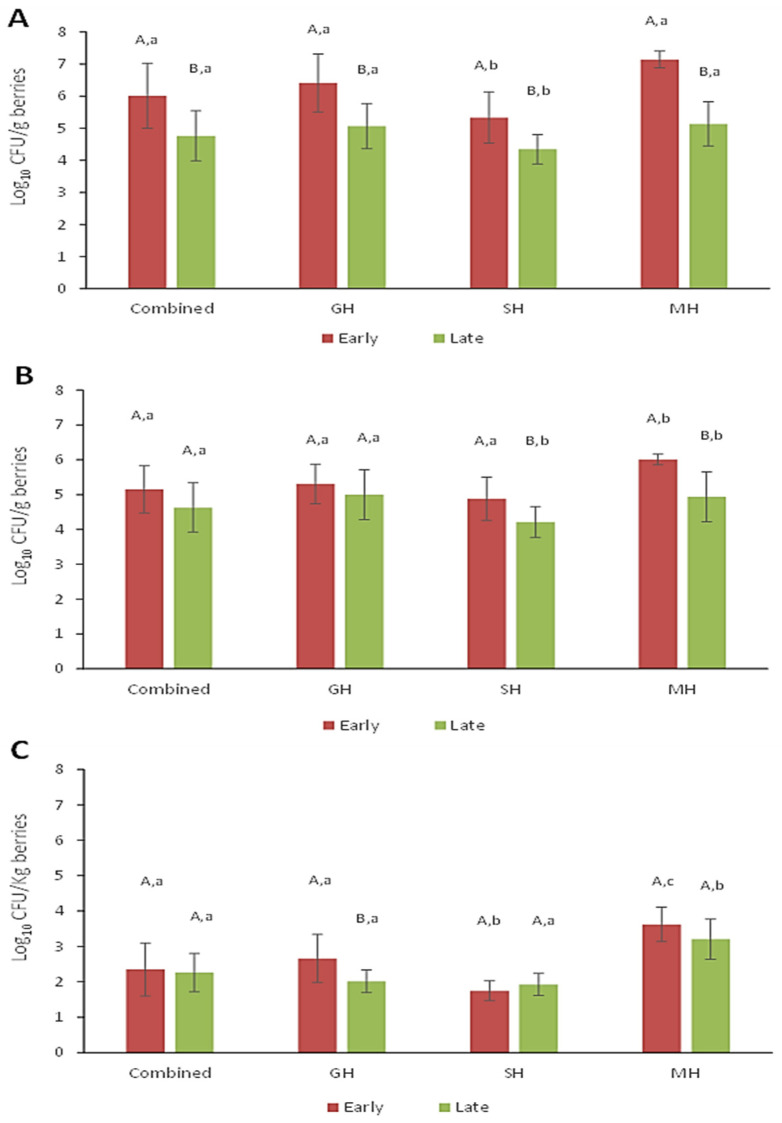
Average counts for (**A**) total aerobic bacteria, (**B**) yeasts and molds, and (**C**) heat-resistant molds on wild lowbush blueberries prior to processing. Counts are delineated by the harvest method (SH, sanitized hand-rakes used by the research team; GH, gently harvested by hand rakes, walk-behind or modified mechanical harvesters; MH, mechanically harvested by traditional tractor mounted harvesters) and time of the season (early versus late). Error bars represent the standard deviations of five composite samples. Different upper-case letters above bars within each harvest method (GH, SH, and MH) indicate a significant difference (*p* < 0.05) in counts between early and late harvest berries. Bars displaying different lower-case letters indicate significant differences between harvest methods within each harvest season (*p* < 0.05).

For APCs, YMCs, and HRM counts, composite samples (n = 70) were prepared by combining equal portions of the homogenates of five individual samples taken from the same field. The results delineated by season (early versus late) and sample type are presented in Figure 1. In general, berries collected during the early part of the harvest season had significantly greater (*p* < 0.001) total numbers of aerobic bacteria as well as yeast and molds. APC values for all sample types combined were more than 1.2 log greater for the early samples (6.01 ± 1.01 log_10_ CFU/g) than those collected later in the season (4.76 ± 0.78 log_10_ CFU/g) (Figure 1A). Although the overall YMC values were ~0.5 log higher for the early season berries over the late season samples (5.15 ± 0.68 versus 4.63 ± 071 log_10_ CFU/g) (Figure 1B), this did not result in significantly different numbers (*p* = 0.82) for HRM between harvest periods (Figure 1C). The type of method used to harvest the berries also had a significant impact on APC and YMC results. Early season samples from MH berries had APCs of 7.14 ± 0.26 log_10_ CFU/g, but these numbers were 2 log lower for berries gathered during the late season (Figure 1A). Similarly, YMCs for MH berries during the early and late season were 6.01 ± 0.15 and 4.94 ± 0.71 log_10_ CFU/g, respectively (Figure 1B). MH berries also had significantly greater numbers of HRM relative to GH or SH-harvested berries (*p* < 0.001). Specifically, HRM numbers (3.6 ± 0.49 log_10_ CFU/Kg) from the early season MH berries were at least 1 log greater than those on GH or SH berries (Figure 1C). MH berries from the late season harvest contained HRMs that were only slightly less prevalent (3.2 ± 0.57 log_10_ CFU/Kg).

In all cases, berries harvested by our research staff (SH) had the lowest APC, YMC, and HRM values relative to all other field samples. Early season values of 5.33 ± 0.80, 4.88 ± 0.62 log_10_ CFU/g, and 1.7 ± 0.28 log_10_ CFU/Kg were observed for APCs, YMCs, and HRM counts, respectively; late season samples displayed significantly lower APC (4.34 ± 0.46 log_10_ CFU/g; *p* < 0.001) and YMC numbers (4.21 ± 0.44 log_10_ CFU/g; *p* = 0.004), where HRM values were unchanged (*p* = 0.256). Overall GH harvested berries (i.e., hand-raked by farm crews, modified mechanical harvesters, or walk-behind harvesters) produced APC and YMC levels intermediate to those obtained for MH and SH berries. In summary, overall microbial loads, especially influenced by aerobic bacteria counts, were significantly greater (*p* < 0.05) on berries harvested during the early part of the season regardless of harvest method. When parsed according to the harvest method, a general descending trend in microbial numbers was observed with MH > GH >SH.

#### 3.1.2. Impact of Farm Practices and/or Location

**Figure 2:**
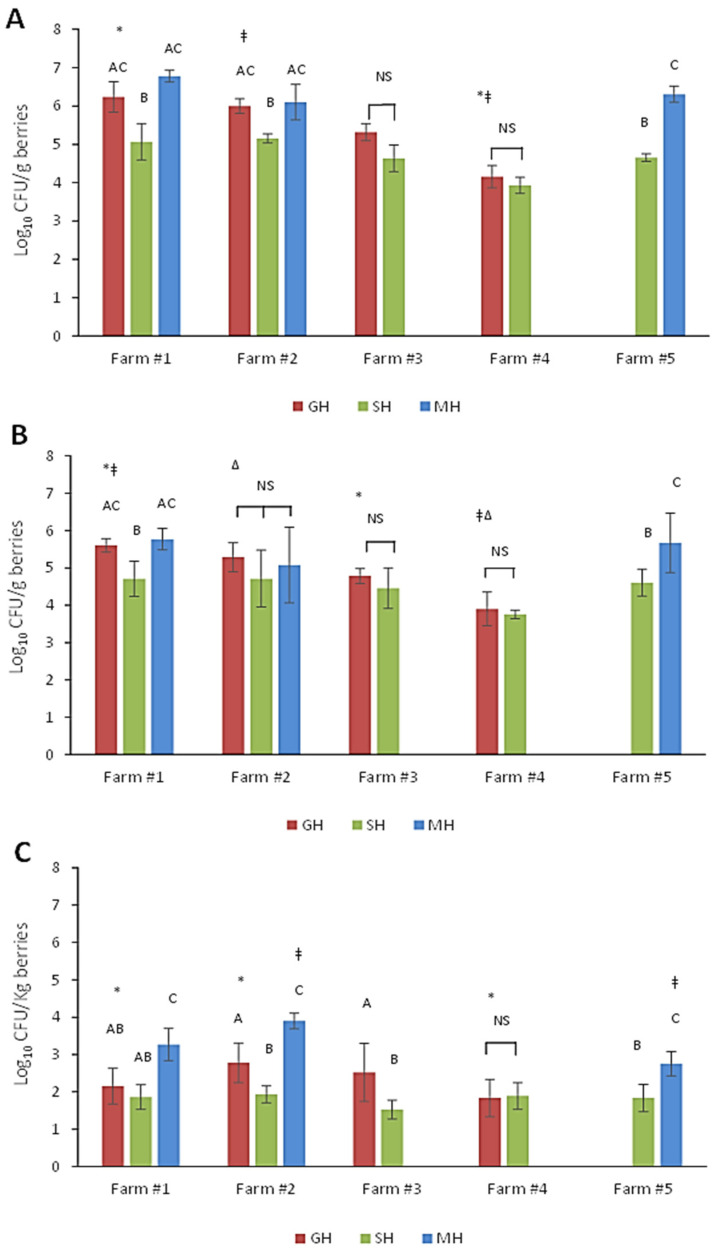
Average counts for (**A**) total aerobic bacteria, (**B**) yeasts and molds, and (**C**) heat-resistant molds on wild lowbush blueberries prior to processing. Counts are delineated by the harvest method (SH, sanitized hand-rakes used by the research team; GH, gently harvested by hand rakes, walk-behind or modified mechanical harvesters; MH, mechanically harvested by traditional tractor mounted harvesters) and participating farm sites. Error bars represent the standard deviations of five composite samples. Different upper-case letters above bars indicate a significant difference (*p* < 0.05) in counts between the harvest methods (GH, SH, and MH) at each farm location. NS = no significant difference. Bars displaying the same symbol (asterisks, alveolar, delta) represent counts that are significantly different (*p* < 0.05) for each harvest method across all farms.

APC, YMC, and HRM levels were also delineated by the farm number and harvesting practices (Figure 2). For Farm #1 the trend of APC and YMC values followed that of the overall combined samples (e.g., MH > GH > SH). The mean APC level for the MH samples was 6.78 ± 0.15 log_10_ CFU/g, while the YMC was 5.77 ± 0.29 log_10_ CFU/g (Figure 2A,B). The alternative GH methods (mainly hand-raked for this location) only marginally decreased APC and YMC numbers to 6.23 ± 0.40 and 5.63 ± 0.18 log_10_ CFU/g, respectively. Those berries harvested by our staff displayed the lowest numbers for both APC (5.07 ± 0.48 log_10_ CFU/g) and YMCs (4.71 ± 0.47 log_10_ CFU/g); levels of which were both significantly less (*p* = 0.048) than for GH berries from this site. HRM levels were also over 1 log greater on MH berries than those obtained by other harvesting methods (Figure 2C). For SH berries collected at Farm#2, the APC levels (5.15 ± 0.12 log_10_ CFU/g) were also statistically significantly less (*p* = 0.041) than those determined for either MH berries (6.10 ± 0.46 log_10_ CFU/g) or GH berries acquired using a modified harvester (6.00 ± 0.19 log_10_ CFU/g). Although YMC values associated with the SH berries (4.71 ± 0.71 log_10_ CFU/g) from Farm #2 appeared to be substantially less than those on GH berries (5.29 ± 0.39 log_10_ CFU/g), it was not statistically significant (*p* > 0.05) due to the wide variations in counts. Unique to this site, YMC levels found on MH berries were lower (5.07 ± 1.01 log_10_ CFU/g) than those on their GH counterparts, albeit large variations in numbers were observed between individual samples. MH samples from this site also produced the highest HRM counts (3.89 ± 0.21 log_10_ CFU/Kg on MH berries) of all samples evaluated at any site. These numbers were approximately 2.0 log greater than those determined for SH berries from the same site.

Traditional mechanically harvested samples (MH) were not collected from Farm #3 or Farm #4 (Figure 2). Although GH Berries from Farm #3 (hand-raked or walk behind harvester) had higher APC (5.32 ± 0.22 log_10_ CFU/g) and YMC (4.78 ± 0.20 log_10_ CFU/g) values than those hand-raked by our staff (SH), the differences were not statistically significant (*p* > 0.05). Overall, mean microbial counts obtained for berries collected from Farm #4 were the lowest observed among all sampling sites. Specifically, the total aerobic bacteria on GH berries were 4.15 ± 0.29 log_10_ CFU/g, a value significantly lower than that found on GH berries from Farm#1 (*p* < 0.001) or Farm #2 (*p* = 0.02), and substantially less than those collected from Farm #3. APC values for SH berries from this site were only 3.93 ± 0.21 log_10_ CFU/g. Although not statistically different (*p* > 0.05), this was lower than all other sites. Similarly, YMC values were 3.90 ± 0.45 and 3.75 ± 0.11 log_10_ CFU/g for GH and SH, respectively, and the HRM contingent on both berry types were nearly the same (1.83 ± 0.50 to 1.88 ± 0.36 log_10_ CFU/kg). Finally, for Farm #5, only MH berries along with those collected by our team (SH) were obtained. APC values for MH samples were 6.31 ± 0.21 log_10_ CFU/g while counts for SH samples were significantly lower (*p* < 0.001) at 4.65 ± 0.10 log_10_ CFU/g. Similarly, YMC values for the MH samples (5.67 ± 0.80 log_10_ CFU/g) were significantly greater (*p* < 0.001) than those obtained by careful hand-raking (SH) (4.61 ± 0.36 log_10_ CFU/g). To summarize, only two of the participating farms used both GH and MH methods (i.e., Farms #1 and #2). Again, there was a general descending trend in overall microbial numbers observed with MH > GH > SH at each site. Total microbial counts on GH berries obtained at these sites were also significantly greater (*p* < 0.05) than those obtained from Farm #3. Compared to the GH and MH berries, the SH berries had the lowest count at each of the five farms. Since microbial numbers on SH berries did not significantly vary (*p* > 0.05) across the farms this indicated that the on-farm practices at each site influenced the variability observed for GH berries.

### 3.2. Presence of Coliforms, Generic E. coli, STEC O157, and Salmonella spp.

**Figure 3:**
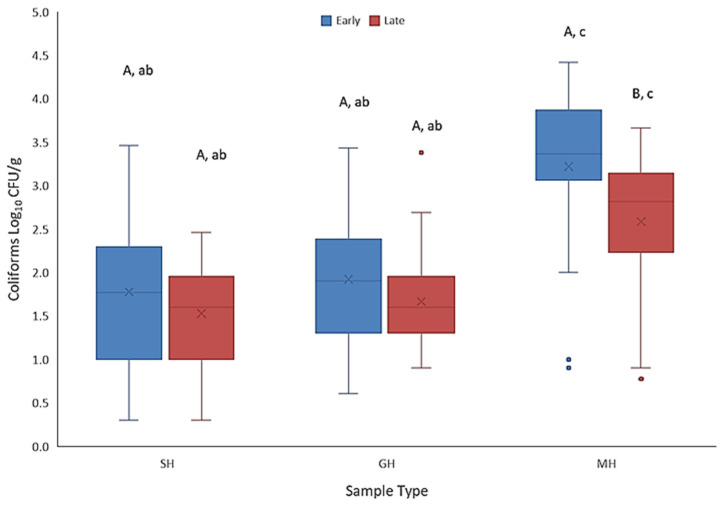
Box plot demonstrating the distribution and density of coliform bacteria on wild lowbush blueberries delineated by harvest time (early versus late season) and method (SH, sanitized hand-rakes used by the research team; GH, gently harvested by hand rakes, walk-behind, or modified mechanical harvesters; MH, mechanically harvested by traditional tractor mounted harvesters). Different upper-case letters above the bars within each harvest method (GH, SH, MH) indicate a significant difference (*p* < 0.05) in counts between early and late-harvest berries. Bars displaying different lower-case letters indicate significant differences between the harvest methods within each harvest season (*p* < 0.05).

All individual wild blueberry samples (n = 350) were screened for the presence of coliforms and more specifically, *E. coli* as indicators of general hygiene and possible fecal contamination, respectively. For these assessments, 3M™ Petrifilm™ *E. coli*/Coliform Count Plates and/or 3M™ Petrifilm™ High-Sensitivity Coliform Count Plates were used. For the hand-raked samples (n = 160) harvested by our research staff (SH), 29.4% of all collected samples tested positive for coliforms, with significantly (*p* < 0.001) more early season harvested samples (42.5%) testing positive than those collected late in the season (16.2%) (Table 2). Coliform-positive samples also varied by field site. For the early harvested berries, 55%, 50%, 50% 20%, and 10% of samples were positive for fields associated with Farm #2, Farm #1, Farm #4, Farm #3, and Farm #5, respectively. Regarding late-harvest SH berries, coliform presence was far less frequent. In fact, no coliform colonies were detected among the 10 samples collected from Farm #4, while only 1 of 20 samples from Farm #2 were positive. For both Farm #3 and Farm #5 the number of positive samples was just 10%. The anomaly to this pattern of low occurrence was Farm #3, where 9 of 10 samples collected tested positive (Table 2). When present, there was also considerable variation in coliform numbers among both early and late-season samples with the overall mean counts of the early berries being significantly greater (*p* = 0.02) than those found on late berries. For example, the early season samples ranged from 0.30 to 3.46 log_10_ CFU/g with a median of 1.78 log_10_ CFU/g. The range for the late harvest barriers had a slightly narrower range (0.30 to 2.46 log_10_ CFU/g) and median value (1.60 log_10_ CFU/g) (Figure 3). Since none of the SH samples tested positive for the presence of *E. coli*, we did not conduct selective enrichments for either STEC or *Salmonella* spp. as the absence of the indicator at the detection limit of the assay suggested the samples were not contaminated with fecal material.

The frequency of coliform presence on berries collected by farm crews using gentle harvesting (GH) methods was significantly greater (*p* < 0.05) than that observed for berries harvested by our staff (SH) (Table 2). Coliforms were detected on 72.9% of all GH berries, with 82.9% of these samples being from the early harvest and 62.9% found among late harvest samples. Although a far greater percentage of samples (relative to SH samples) tested positive for coliforms, their actual numbers remained low with comparable ranges to the SH samples (Figure 3). The range for coliform-positive early harvest samples was 0.60 to 3.43 log_10_ CFU/g with a median of 1.90 log_10_ CFU/g, whereas for late season samples, the range was 0.90 to 2.69 log_10_ CFU/g with an identical median to their SH counterparts (i.e., 1.60 log_10_ CFU/g). Again, coliform-positive sample numbers varied by the farm from which they were acquired. All samples for early harvest berries from Farms #3 and #4 tested positive, while 83% and 65% of samples were positive for samples from Farm #1 and Farm #2, respectively. For late-harvest GH berries, coliforms were detected on 95%, 90% and 53.3% of berries from Farms #2, #3 and #1. However, none of the ten samples collected from Farm #4 tested positive (Table 2). Regarding the presence of potential fecal contamination among samples that tested positive for coliform bacteria, three of these samples also tested positive for *E. coli*. Two 3M™ Petrifilm™ *E. coli*/Coliform Count Plates from the initial 10-fold sample dilution (berry homogenate) presented a single *E. coli* colony while two colonies were present on a third plate also from the original homogenate. All of these samples were from Farm #1 during the early season harvest. All *E. coli*-positive samples were further processed for the carriage of human pathogens. However, the downstream enrichments for the specific detection of either STEC O157 or *Salmonella* spp. did not indicate the presence of these human pathogens.

Berries harvested by traditional mechanized harvesters generally accumulate considerably more debris (e.g., soil, leaf litter, etc.) than GH berries. As such, 92% of samples from either the early or late harvest tested positive for coliforms (Table 2). Again, the range was broad but the overall load in both the early and late-harvest samples was significantly greater (*p* = 0.005) than that found in the SH and GH berry samples. Moreover, coliform numbers on early harvest MH berries were also significantly higher (*p* < 0.001) than on their late-harvest counterparts. The highest levels for early MH samples were found to be 4.41 log_10_ CFU/g and the median was 3.36 log_10_ CFU/g which were significantly greater (*p* < 0.001) than those observed on the SH or GH samples. As for late-season MH berries, the associated coliform numbers were also significantly more (*p* < 0.001) than on the late SH and GH berries (maximum, 3.66 log_10_ CFU/g; median, 2.82 log_10_ CFU/g). In total, 100% of all samples (both early and late season) collected from Farm #1 and Farm #5 were positive for coliforms, whereas MH samples from Farm #2 for both seasons were 80% positive for coliforms. *E. coli* was detected on 18% of MH samples, with five coming from Farm #5 (early) and four from Farm #1 (late). The mean *E. coli* count for Farm #5 samples was 2.73 log_10_ CFU/g, while for Farm #1 the average was 1.93 log_10_ CFU/g. All coliform-positive samples were enriched for STEC O157 and *Salmonella* spp. but neither pathogen was detected. In summary, the frequency of coliform presence in the berry samples and their associated counts were largely correlated to high aerobic plate counts. Compared to traditional MH methods, samples collected using the GH or SH methods resulted in fewer coliform-positive samples and lower counts when present. As such, no SH samples and relatively few GH samples (2.1%) were found to contain *E. coli*, whereas this fecal indicator was present in 18% of MH samples. Neither of the human pathogens screened for in this study were found in any of the berry samples.

### 3.3. General Hygiene Assessment

**Figure 4:**
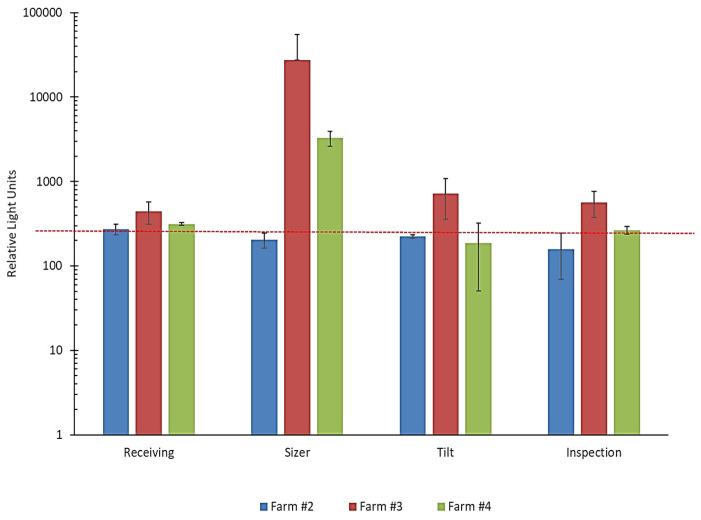
Residual microbial contamination of the conveyor belts on the blueberry processing lines following sanitation procedures. Conveyor belt cleanliness at different junctures of the processing line was assessed by taking surface swabs and measuring ATP activity using the 3M™ CleanTrace™ system. ATP activity is expressed as relative light units (RLUs). The dashed line indicates the recommended cut-off reading for “clean” surfaces. Error bars represent the standard deviation of five readings each for two independent sample periods.

ATP swabs were taken from various conveyor belt surfaces following the cleaning of the food processing lines at three different facilities. The belts were cleaned and sanitized by the site crews in the evening and swabbing was performed the next morning prior to the start of operations. According to the device manufacturer (3M Canada, London, ON, Canada), acceptable ATP levels associated with clean surfaces should be ≤250 Relative Light Units (RLUs). Swab samples taken from the conveyor belts at four different positions along the process lines at Farm #2 showed ATP levels to be below or just slightly above this RLU limit (Figure 4). In contrast, the average RLU readings from the swab samples acquired from Farm #3 exceeded the specified limit at all positions on the processing line. The average reading for the receiving area belt was 444 RLUs while the readings from the “Tilt” and “Inspection” belts were 721 and 567 RLUs, respectively. The swabs taken from the “Sizer” belt at this site produced substantially higher RLUs compared to all other samples with average readings just above 27,000 RLUs. For Farm #4, the belt swabs recovered from the processing line resulted in readings that showed cleaning practices were effective at two locations (i.e., Tilt and Inspection belts). Also, ATP levels determined for Receiving belt swabs were slightly above acceptable values, with mean RLUs of 313. However, the Sizer belt also appeared to be a problem area at this site as average readings of >3200 RLUs were observed. The overall ATP results showed that only the cleaning and sanitizing practices employed by Farm#2 resulted in acceptable readings in all swabbed areas of their processing line. For Farms #3 and #4, the Sizer belt appeared to be a common, hard-to-clean area. More attention needs to be given to all areas of the line at Farm #3.

### 3.4. Detection/Enumeration of L. monocytogenes on Berry Contact Surfaces

A total of 61 surfaces from the processing line conveyor belts (n = 31) and blueberry totes (n = 30) were swabbed to assess their carriage of *L. monocytogenes*. Following Fraser broth enrichments and plating onto both modified Oxford selective agar and RLM agar, four swab samples (3 belts, 1 totes) revealed colonies with morphologies characteristic of *L. monocytogenes* (Table 3). All three positive belt swabs were from the same farm (Farm #1), two of which were taken during the early harvest season while the third was a mid-season sample. However, when the *Listeria*-positive swab homogenates were plated directly onto selective agar without pre-enrichment, no *Listeria* colonies were present, which implied that the pathogen was present at very low levels. As for the totes, only a single sample recovered from Farm #4 tested positive for *L. monocytogenes* following enrichment but again, numbers were too low to enumerate via direct plating. Although actual numbers of *L. monocytogenes* were low, the presence of the pathogen in 9.6% of the swab samples from the conveyor belts is surprising and could pose a potential health risk.

## 4. Discussion

Wild lowbush blueberries have long been marketed for their high nutritional value and overall healthy goodness. In addition to providing an excellent source of fiber (~2.5 g per 100 g of berries), blueberries are also a good source of manganese, vitamin C, and niacin [23]. Perhaps most notably, wild blueberries have been promoted for their high levels of polyphenols such as anthocyanins and proanthocyanidins, which are thought to improve cardiovascular health [24], while results from other studies have implied that wild blueberries may have a protective capacity against oxidative damage to DNA [25]. Despite these healthy benefits, few studies have examined the potential microbiological hazards associated with wild lowbush blueberries. This lack of research may be partly due to the low frequency of cases of foodborne diseases or food recalls linked to wild blueberries [6]. Whereas other berry types such as strawberries and raspberries have been implicated in numerous outbreaks of foodborne diseases [4,5], the lower incidence for blueberries could in part be attributed to differences in their surface topography which impacts the ability of pathogens to colonize. For example, compared to the surface of raspberries, which contain an abundance of hair-like projections, the blueberry surface is relatively smooth and waxy [26]. Another contributing factor to the infrequency of disease incidents related to wild blueberries could be related to the fact that most of the crop is subjected to rigorous cleaning and washing steps during IQF processing, which has been shown to significantly reduce bacterial numbers, including the frequency of fecal indicators [13].

To the best of our knowledge, the current study is the first to examine the general microbiologic status of wild blueberries harvested in Nova Scotia. The general microbial load (APC, YMC, and HRM) was determined as a measure for the shelf-life potential of the product, while coliform counts and the detection of *E. coli* served as indicators of overall hygiene and the possible presence of fecal contamination, respectively. Factors impacting the quality of wild blueberries are multifaceted and include the harvest method, weather conditions (temperature, humidity) affecting fruit development and at the time of harvest, intrinsic plant features (height and density, clonal type, etc.), and field characteristics (soil moisture, topography, and weed integration) [27]. These same factors in addition to the presence of wildlife in the area, can also play a role in the occurrence and abundance of various microflora associated with wild blueberry soils, foliage, and ultimately, the berries themselves [18].

Berries collected during the first two weeks of the 2021 harvest season (i.e., early) had much higher microbial loads (APCs + YMCs) than those from the late season, irrespective of sample type (GH, MH, and SH). For all samples combined, this difference in mean log_10_ CFU/g was more than 1.2 log for aerobic bacteria and approximately 0.5 log for yeast and molds. Not surprisingly, the highest microbial loads were found on berries harvested by traditional tractor-mounted harvesters (MH). These increased levels can be attributed to greater amounts of sod, soil particulates, leaf litter, and other debris co-collected with the berries. Additionally, rough handling of the berries by such harvesting methods can cause bruising and breaches in the skin, thus resulting in the leakage of juices that could serve as nutrients for the resident microflora [28,29]. Early harvest samples were found to carry as much as 7.1 and 6.0 log_10_ CFU/g of aerobic bacteria and yeast and molds, respectively, whereas APC and YMC levels on the late harvest samples were 5.1 and 4.9 log_10_ CFU/g. The same trend was observed for GH berries, which comprised a combination of hand-rake harvesting (farm staff) and modified machine-harvesting practices. In this case, APCs and YMCs for the early harvested berries were substantially less than those found on MH berries; however, values determined for late harvest GH berries were strikingly similar to those on MH berries. These latter numbers are in range with an earlier study by Hazen et al. (2001) who reported initial APCs of 4.8 log_10_ CFU/g on wild blueberries entering the processing line [13]. Recently, Holland and co-workers (2021) demonstrated that various surfaces on mechanical harvesters harbored elevated levels of total aerobic bacteria, as well as yeasts, and molds [30]. The authors also provided evidence that passage through the harvesters translates to significantly more microorganisms on the berries entering the processing lines.

In comparison, berries carefully harvested by our team using sanitized hand rakes (SH) averaged APC and YCM levels around 5 log_10_ CFU/g during the early harvest and were approximately 1 log lower than that later in the season. The reason for this early versus late season difference is not entirely clear, but we speculate that the high microbial loads may be related to slower or insufficient removal of field heat in early berries. It was noted that daytime temperatures and humidity were excessively high during the first two weeks of sampling with the highest humidex readings exceeding 40 °C on several days [31]. Warmer berries placed into portable coolers containing ice packs may not have been able to dissipate heat, so samples may have remained at relatively warm temperatures for an extended period before being transferred to the laboratory. Moreover, MH berry samples were directly collected from totes in the field, which at times could be left sitting at warm temperatures for long periods before being transported to processing facilities. In another study involving highbush blueberries [16], APC and YMC values were found to be similar to those observed in the present work for SH berries. The authors also noted that berries collected at noon had significantly higher counts than those obtained either in the early morning or late in the day, which supports our theory regarding field heat [16]. Previously, Jackson et al. (1999) showed that minimizing delays in cooling blueberries after harvest resulted in reduced numbers of spoilage microorganisms during refrigerated storage [32]. Quansah et al. (2019) also reported significant differences in microbial counts on berries among the six participating packing houses in their survey [16]. When delineating our data by each farm site, significant differences were also observed. Variations in microbial numbers between samples could be influenced by a combination of extrinsic or intrinsic factors such as climate conditions before and during harvest, ecology of a particular field, and operational practices specific to these sites (e.g., harvest method).

While total microbial loads can serve as good predictors for shelf-life potential, total coliforms and fecal coliforms (*E. coli* specifically) can be used to assess the hygienic quality of foods and the potential for harborage of pathogenic microorganisms [33]. The frequency and abundance of these indicators in the samples collected in our study largely paralleled the trends for total microbial loads. That is, the probability of a sample containing coliforms including *E. coli* increased when APCs were elevated. In the SH sample group (lowest APCs), only 25% of the samples contained coliform bacteria and when present, mean counts were about 2 log_10_ CFU/g. Again, seasonal differences were observed with 35% detection in samples during early harvest and only 15% for late harvest. None of the samples were found to contain *E. coli*. A dramatic increase was noted for berries harvested by farm crews, either by hand or with machinery, with coliform presence on 71% of early harvest and 61% of late harvest berries. Still, the actual counts remained low (mean ~2.5 log_10_ CFU/g). In Canada, the limit for total coliforms is <1000 CFU/g for a number of ready-to-eat (RTE) foods, but fresh fruits and vegetables are exempt from this rule since high numbers can be common due to cultivation practices [34]. Lowbush blueberries grow in close proximity to the ground; thus, soil microflora can be easily deposited on the fruit [6,30]. Machine harvesters also exacerbate this issue since the topography of fields is often uneven. This was especially demonstrated with traditional mechanical harvesters as 92% of 50 samples recovered tested positive for coliforms and at high levels. However, MH berries would be IQF processed, which effectively aids in the reduction in the microbial load [13]. Therefore, growers should take care to avoid contact with the ground when harvesting berries for the fresh-packed market. Although data demonstrated that modified mechanical harvesters reduced the microbial loads relative to traditional tractor-mounted harvesters, careful hand-raking was the superior method. Unfortunately, harvesting wild blueberry fields by hand-raking is labor-intensive, requiring large crews of dependable well-trained personnel, which may not be feasible for growers. Alternatively, practices involving modified mechanical harvesters could be improved to reduce such contact and subsequent buildup of microorganisms on equipment. If equipment is left soiled for extended periods, this could permit the formation of biofilms which could act as a source for pathogen transfer [35]. Periodic pressure washing and sanitizing of this equipment in the field during harvest could be a solution to this issue. However, harvesters would need to be dried before harvesting resumes.

In contrast to the ambiguity for acceptable coliform levels on fresh produce, limits for generic *E. coli*, when detected, are better defined, where satisfactory, marginal, and unsatisfactory levels (i.e., CFU/g) are set at <10, 10 to 99, and ≥100, respectively [34]. Of 12 samples (3.4%) that tested positive for *E. coli*, 3 were GH early-harvest samples from a single farm, while the other 9 were associated with MH samples from two different farm sites. In all cases, the positive samples occurred on the same sample day for their respective sites. Only early season MH samples were observed to exceed the *E. coli* limit with average counts of 2.7 log_10_ CFU/g), and it was these same samples that correlated with high coliform numbers (4.3 log_10_ CFU/g). No samples were found to contain either STEC O157 or *Salmonella* spp., despite the presence of generic *E. coli*. The significantly greater loads of aerobic bacteria observed on the early harvest berries factored into the high coliform numbers and subsequently higher probability of encountering *E. coli.* The few other studies examining the microbiology of wild blueberries provided similar results in that the carriage of human pathogens appears to be sporadic [16,30]. Still, outbreaks of enteric bacterial pathogens have been linked to blueberries in the past. For example, blueberries contaminated with *Salmonella* Muenchen in 2009 resulted in 14 illnesses [10], while a year later blueberries were implicated in an outbreak of *Salmonella* Newport in northwestern Minnesota [36]. Therefore, it is important that good manufacturing practices are in place to mitigate such risks.

The harvest season for wild blueberries in Nova Scotia is relatively short (5–6 weeks). Processing lines for fresh market berries frequently operate continuously throughout each day without undergoing thorough sanitization and cleaning as wetting needs to be avoided. Therefore, they can be subject to the buildup of fine debris and residues that can trap or allow for the colonization of microorganisms. This could lead to areas that may be difficult to completely sanitize once operations cease. Consequently, this puts greater emphasis on the cleanliness of berries entering processing facilities. Reducing soil debris and other particulates entering the line would help reduce available microbial numbers that could colonize surfaces, especially hard-to-clean areas [26]. Environmental ATP swab readings demonstrated that the conveyor belts associated with the sizer section on two of three processing lines were problematic. In the study by Gazula et al. (2019), the sizer units did not appear to be an issue for the processors involved [15]. However, in the latter study, highbush blueberries were being processed so one would not expect a high degree of soil-related debris entering these lines. Future work needs to be conducted to elucidate the reasons for these observations.

Although ATP monitoring provides a good indication of general cleanliness along the processing line, we gauged the potential for pathogen biofilms if good manufacturing practices (GMP) are not followed. Therefore, environmental swabs from the conveyor belts and field totes were collected before cleaning from four farms at various points over the season. The opportunistic intracellular pathogen *L. monocytogenes* is widespread in nature and known for its ability to survive and persist for long periods in food production areas [37]. Of the 61 environmental sponge/swab samples (31 belts and 30 totes) tested for *L. monocytogenes*, 4 were positive for the pathogen. This included three belt swabs from a single farm and the other from a tote from another farm. It is worth noting that two of the three positive belt samples were taken at separate times during the same day (i.e., early morning and noon), while the third came from swabbing belts on a different day. In all cases, *Listeria* was only detectable in enrichment cultures as direct plating the swab homogenates onto selective media, produced no *Listeria* colonies, thus indicating the pathogen numbers were low. Since the acidic nature of wild blueberries would prevent the growth of *L. monocytogenes*, one could surmise that substantial contamination of berries would have to occur by contacting surfaces containing elevated levels of the pathogen to be of concern [38]. However, although growth is not expected, *L. monocytogenes* can survive on blueberries during refrigerated storage or when frozen [39]. This may pose a risk for consumers as blueberries are often added as ingredients in other foods, such as salads and smoothies, which could result in a more favorable environment for *Listeria* growth if stored too long at permissive temperatures [40]. Therefore, blueberry processors should apply effective programs for cleaning and sanitizing contact surfaces [41,42] and for monitoring their products for contamination with *L. monocytogenes* and other foodborne pathogens. Consumers can exercise due diligence as well by thoroughly washing berries in hot water prior to consumption. One study showed that microbial counts on highbush blueberries dipped in hot water (60 °C) for 30 s could be reduced by 0.7 log [43].

This study examined the microbiological status of berries entering processing lines with particular interest in potential contamination with fecal material. Also, to a lesser extent, cross-contamination of berry-contact surfaces was investigated via environmental swab sampling of totes and conveyor belts. Since incoming berries were shown to have relatively high levels of aerobic bacteria and sporadically harbor fecal indicator organisms, future research should focus on the end products to ascertain the impact of passage through the processing line on microbial loads. Moreover, results also demonstrated that approximately 5% of environmental swab samples taken from conveyor belts were contaminated with *L. monocytogenes*. Therefore, downstream studies should include more intensive environmental sampling to better assess the presence of this pathogen and determine potential “hot spots” along processing lines. A metagenomics approach could be employed to better understand the microorganisms associated with the berries and their relationship to human pathogens [44].

## 5. Conclusions

Herein, we examined the microbiological status of Nova Scotia lowbush blueberries during the 2021 season. The microbial load associated with a particular sample of blueberries can vary widely between fields and even within the same field. Our results showed that the time of harvest season, environmental and climatic factors, the harvesting method, and field location impact the microbial load on the berries. As such, the frequency of coliform presence in the berry samples could generally be associated with higher levels of aerobic bacteria. Harvesting methods that minimized contact with the ground (i.e., sanitized hand-raking) resulted in significant reductions in microbial numbers and the frequency of detection of coliforms as well as their levels. Therefore, producers should take these factors into consideration when designating berries for the fresh-packed market. Although our results imply that the risk for carriage of enteric pathogens is low, sporadic occurrences of fecal indicators on incoming berries, as well as the detection of *L. monocytogenes* on swab samples from berry contact surfaces, shows that the risk of pathogen contamination is possible. Therefore, for fresh-packed berries, growers should (1) select appropriate harvest sites with relatively flat topography, (2) pursue harvest methods that better reduce the level of soil debris entering the processing lines, (3) minimize post-harvest exposure to warm temperatures, (4) ensure regular and effective sanitation of the processing line, and (5) maintain vigilance by screening for pathogens such as *L. monocytogenes*. It would also be prudent to seek novel processing innovations aimed at enhancing the safety of their products. For example, Hassani et al. (2020) described the optimization of a vapor-phase advanced oxidation process (AOP) to treat table grapes [45]. This innovation, which employs a combination of UV-C, ozone, and H_2_O_2_, was demonstrated to reduce levels of *L. monocytogenes* by 4–5 log. The implementation of end-point anti-microbial treatment systems would not only ensure the safety of fresh-packed blueberries but also extend the shelf-life of these products. In addition, the wild blueberry industry should continue to support research to help better understand the relationship between typical resident microflora found on wild lowbush blueberries (and processing environments) and foodborne pathogens such as *Listeria monocytogenes*. Knowledge in this area could help bridge this gap and mitigate food safety issues in the future.

## Figures and Tables

**Table 1:** Sampling scheme for microbiological assessment of pre-processed wild lowbush blueberries.

		Fields or Lots Sampled per Farm							
Sample Type	Season	Farm #1	Farm #2	Farm #3	Farm #4	Farm #5	Total Fields or Lots	Samples per Field or Lot	Total
Field (harvested by research team with sanitized hand rake)	Early	6	4	2	2	2	16	5	80
	Late	6	4	2	2	2	16	5	80
Gently harvested (hand rake, walk behind, or modified mechanical)	Early	6	4	2	2	0	14	5	70
	Late	6	4	2	2	0	14	5	70
Traditional mechanical tractor mounted harvester	Early	2	2	0	0	1	5	5	25
	Late	2	2	0	0	1	5	5	25
TOTAL									350

**Table 2:** Coliform counts and pathogen presence in blueberry samples harvested by different methods. For each bacterium, the numbers indicate the total number of samples that tested positive with the corresponding percentage of samples given in parentheses.

Season	Farm #	Total Fields	Total Samples	Positive Coliforms (%)	Positive *E. coli* (%)	Positive STEC	Positive *Salmonella*
SH—hand-harvested using sanitized rake (Research staff)							
Early	1	6	30	15 (50.0)	0	0	0
	2	4	20	11 (55.0)	0	0	0
	3	2	10	2 (20.0)	0	0	0
	4	2	10	5 (50.0)	0	0	0
	5	2	10	1 (10.0)	0	0	0
TOTALS		16	80	34 (42.5)	0	0	0
Late	1	6	30	3 (10.0)	0	0	0
	2	4	20	1 (5.0)	0	0	0
	3	2	10	9 (90.0)	0	0	0
	4	2	10	0	0	0	0
	5	2	10	1 (10.0)	0	0	0
TOTALS		16	80	13 (16.0)	0	0	0
CUMULATIVE		32	160	47 (29.4)	0	0	0
GH—gently harvested (Farm crew: hand-raked, modified mechanical or walk behind harvester)							
Early	1	6	30	25 (83.0)	3 (10.0) ^a^	0	0
	2	4	20	13 (65.0)	0	0	0
	3	2	10	10 (100)	0	0	0
	4	2	10	10 (100)	0	0	0
TOTALS		14	70	58 (82.9)	3 (4.3)	0	0
Late	1	6	30	16 (53.3)	0	0	0
	2	4	20	19 (95.0)	0	0	0
	3	2	10	9 (90.0)	0	0	0
	4	2	10	0	0	0	0
TOTALS		14	70	44 (62.9)	0	0	0
CUMULATIVE		28	140	102 (72.9)	3 (2.1)	0	0
MH—mechanically harvested (Traditional tractor mounted)							
Early	1	2	10	10 (100%)	0	0	0
	2	2	10	8 (80.0%)	0	0	0
	5	1	5	5 (100%)	5 (100) ^b^	0	0
TOTALS		5	25	23 (92.0%)	5 (20.0)	0	0
Late	1	2	10	10 (100%)	4 (40.0) ^c^	0	0
	2	2	10	8 (80.0%)	0	0	0
	5	1	5	5 (100%)	0	0	0
TOTALS		5	25	23 (92.0%)	4 (16.0)	0	0
CUMULATIVE		10	50	46 (92.0%)	9 (18.0)	0	0

Mean *E. coli* counts: ^a^ ≤10 CFU/g, ^b^ 8.5 × 10^1^ CFU/g; ^c^ 5.36 × 10^2^ CFU/g.

**Table 3:** Detection of *L. monocytogenes* on blueberry processing contact surfaces.

Farm #	Season	Belts	*L. monocytogenes* Positive	Totes	*L. monocytogenes* Positive
	Early	4	2 *	3	0
1	Middle	3	1 *	3	0
	Late	3	0	3	0
	Early	3	0	3	0
2	Middle	1	0	1	0
	Late	2	0	0	0
	Early	2	0	4	0
4	Middle	0	-	0	0
	Late	3	0	3	1 *
	Early	4	0	3	0
5	Middle	3	0	3	0
	Late	3	0	3	0
TOTALS		31	3	30	1

* No *Listeria* detected by direct plating 1 mL of homogenate onto modified Oxford agar.

## Data Availability

Data will be made available upon request.
